# Energetic Plasticity Underlies a Variable Response to Ocean Acidification in the Pteropod, *Limacina helicina antarctica*

**DOI:** 10.1371/journal.pone.0030464

**Published:** 2012-04-20

**Authors:** Brad A. Seibel, Amy E. Maas, Heidi M. Dierssen

**Affiliations:** 1 Biological Sciences, University of Rhode Island, Kingston, Rhode Island, United States of America; 2 Biological Sciences, University of Rhode Island, Kingston, Rhode Island, United States of America; 3 Marine Sciences, University of Connecticut, Groton, Connecticut, United States of America; National Oceanic and Atmospheric Administration/National Marine Fisheries Service/Southwest Fisheries Science Center, United States of America

## Abstract

Ocean acidification, caused by elevated seawater carbon dioxide levels, may have a deleterious impact on energetic processes in animals. Here we show that high PCO_2_ can suppress metabolism, measured as oxygen consumption, in the pteropod, *L. helicina forma antarctica*, by ∼20%. The rates measured at 180–380 µatm (MO_2_  = 1.25 M^−0.25^, p = 0.007) were significantly higher (ANCOVA, p  =  0.004) than those measured at elevated target CO_2_ levels in 2007 (789–1000 µatm,  =  0.78 M^−0.32^, p  =  0.0008; Fig. 1). However, we further demonstrate metabolic plasticity in response to regional phytoplankton concentration and that the response to CO_2_ is dependent on the baseline level of metabolism. We hypothesize that reduced regional Chl *a* levels in 2008 suppressed metabolism and masked the effect of ocean acidification. This effect of food limitation was not, we postulate, merely a result of gut clearance and specific dynamic action, but rather represents a sustained metabolic response to regional conditions. Thus, pteropod populations may be compromised by climate change, both directly via CO_2_-induced metabolic suppression, and indirectly via quantitative and qualitative changes to the phytoplankton community. Without the context provided by long-term observations (four seasons) and a multi-faceted laboratory analysis of the parameters affecting energetics, the complex response of polar pteropods to ocean acidification may be masked or misinterpreted.

## Introduction

Anthropogenic carbon dioxide (CO_2_) diffuses into the ocean causing a reduction in pH. This “ocean acidification” may have a deleterious impact on energetic processes, including calcification, growth and metabolism, in marine organisms [1]–[3]. Thecosomatous pteropods, in particular, are widely believed to be susceptible to ocean acidification due to their fragile shells made of aragonite, a highly soluble form of calcium carbonate [4], [5]. However, an organism’s nutritional state and feeding history also influence energetic parameters. Low regional phytoplankton concentrations, for example, have been implicated in reduced population abundance, delayed spawning, metabolic suppression and local extinction [6], [7] in Antarctic pteropods. Moreover, phytoplankton themselves are known to be sensitive to CO_2_ in some cases [8]. Thus ocean acidification may impact pteropods both directly and indirectly via changes to the phytoplankton community.

The physiological challenges associated with ocean acidification stem from the decreased outward gradient of carbon dioxide from the cells to seawater. Because CO_2_ reacts with intra- and extracellular fluids just as it does with seawater, internally elevated levels may cause a respiratory acidosis [1]–[3]. Most organisms have some capacity to control internal acid-base status via buffering and ion transport, but there is an associated energetic cost that may be responsible for the trade-offs sometimes observed in the response to hypercapnia [9]. Recent evidence suggests that available energy plays a large role in the response of animals to ocean acidification and that enhanced nutrition can ameliorate the effects in some cases [10]. Hence, an increase in the rate of metabolism may be expected with ocean acidification, given adequate energy availability [11].

Alternatively, internal acidosis and environmental hypercapnia are known to trigger metabolic suppression in some organisms [12], [13]. Such suppression is an intrinsic, adaptive strategy to extend survival time during exposure to short-term hypercapnia, hypoxia, or food deprivation in many organisms [12]. Food limitation and elevated CO_2_ co-occur in winter in the Southern Ocean because light limits productivity and the concomitant drawdown of CO_2_ from surface waters [14]. Both of these parameters may alter energy budgets in marine animals [15], [16]. Metabolic suppression is typically achieved by shutting down expensive processes, such as protein synthesis and ion transport [12], which is obviously not advantageous under chronic stress. Reduced protein synthesis will, by definition, reduce growth and reproductive potential. While suppression of metabolism is, under most experimental conditions, a “sublethal” reversible and adaptive process, reductions in growth and reproductive output will have deleterious impacts on the species at a population level when sustained over longer time scales as may be expected under chronic ocean acidification.

Our study presents annual variation in rates of metabolism measured in the shelled Antarctic pteropod *Limacina helicina forma antarctica* (hereafter called *L. antarctica*) collected from McMurdo Sound. Physiological rates measured in the lab were also related to remotely sensed phytoplankton abundance (Chl *a* levels) in the local environment, a proxy for food availability [6], [7]. We tested the consistency of metabolic rates and the dependence of those rates on environmental variability over four field seasons (January of 1999, 2000, 2007, and 2008). We evaluate the utility of common experimental approaches that are used to assess the ecological impact of ocean acidification and conclude that elevated carbon dioxide does result in metabolic suppression in *L. antarctica*, but that plasticity in baseline levels of metabolism can confound and mask this effect.

## Methods

Specimens of *Limacina antarctica* were found along ice-free shores of Cape Byrd, Cape Evans and Cape Royd on Ross Island, McMurdo Sound, Antarctica. No permits or specific permissions were required for this work in these locations and the study site is not privately owned. *Limacina antarctica* is not an endangered or protected species. Individuals were collected between January 5 and February 8 in 2007 and 2008 by hand using “jelly dippers” (beakers attached to the end of a broom handle) and were maintained at densities of 10 l^−1^ in environmental rooms at McMurdo Station, Antarctica until acclimation. A subset of specimens were held in food-deprivation trials in filtered seawater in large static chambers at densities of 5 l^−1^ prior to acclimation. All other specimens were held for less than two days after capture and prior to acclimation. Following capture and, in some cases, food deprivation, specimens were acclimated at densities < 1 l^−1^ for 24 hours in seawater bubbled with certified gas mixtures containing variable CO_2_ concentrations (Table 1, see below).

**Table 1:** Oxygen consumption rates (MO_2_, µmoles O_2_ g^−1^ h^−1^) of *Limacina helicina antarctica* in relation to carbon dioxide treatments presented as means and also normalized to a common body mass of 5 mg assuming a scaling coefficient (*b*, MO_2_  =  b_0_M*^b^*) of −0.25.

Year	Chl a, mg m^−3^ (±SD)	PCO_2_ µatm	*n*	Size Range (mg)	Mean (±SD)	5 mg (±SD) *b* = −0.25
**1998–99**	3.55 (±3.11)	380	12	1.5–5.0	5.51 (1.52)	5.20 (±1.39)
**2000–01**	0.85 (±1.14)	380	21	2.0–17.2	3.78 (0.75)	3.42 (0.88)
**2006–07**	3.56 (±3.92)	180	7	3.8–7.5	4.91 (0.81)	4.99 (0.67)
		380	15	2.4–14.9	4.47 (0.90)	4.76 (1.27)
		790	7	2.5–14.1	3.48 (0.82)	3.94 (0.80)
		1000	8	3.1–11.0	4.37 (0.93)	4.31 (0.92)
		1500	5	5.9–9.6	3.39 (0.47)	3.76 (0.40)
**2006–07**	(lab starved)	180	7	4.4–12.8	3.19 (0.43)	3.43 (0.66)
		380	13	3.4–13.5	3.35 (0.77)	3.82 (0.77)
		560	8	4.2–7.6	3.61 (0.93)	3.76 (1.01)
		790	8	3.5–10.3	3.37 (0.65)	3.62 (0.67)
		1000	7	3.5–10.0	3.14 (0.54)	3.24 (0.48)
		1800	8	2.1–8.3	3.38 (0.51)	3.52 (0.51)
**2007–08**	1.60 (±2.90)	380	41	0.8–10.5	4.21 (2.04)	3.34 (1.43)
		1000	34	1.2–14.4	3.43 (0.98)	3.07 (0.95)

Chlorophyll *a* concentrations are also shown.

The seawater temperature in McMurdo Sound varies from about −1.7 to −0.5°C in January [17] and the animals were maintained in the lab and in experiments at −1.8°C. No PCO_2_ data were collected at the sites of animal collection but studies indicate seasonal variability with lower PCO_2_ levels during austral summer when phytoplankton blooms take up CO_2_ [18]. Target CO_2_ concentrations for our experiments were 180, 280, 380, 560, 790, 1000, 1500 and 1800 µatm. Total total alkalinity (TA) and pH (total scale) were measured optically according to the best practices guide for ocean acidification research [20] for the most common gas concentrations used (380, 790 and 1000 µatm). The pH was calculated from voltage readings calibrated using certified reference material (CRM) with a known pH (Prof. A. Dickson, Scripps Institution of Oceanography, La Jolla, California) as standard. The dissolved inorganic carbon, PCO_2_ and CO_3_^ = ^ concentration were calculated using CO_2_ sys (Table 1) [21].

Food deprivation trials were conducted for up to 13 days following capture. Oxygen consumption rates were measured in groups of specimens each day of the trial. The starved individuals (Table 2) are those that were held for 4–6 days post capture prior to measurement. They are compared to specimens held for less than 2 days post capture. No change in metabolism was apparent between 4 and 13 days post-capture [6]. All specimens were alive and swimming actively following the food-deprivation trial.

**Table 2:** Experimental seawater carbonate chemistry at target gas levels.

Target	Mean PCO_2_ (ppm ± SD)	Mean pH (± SD)	Mean TA (µmoles ± SD)	Aragonite Saturation
**380**	372 (24)	8.071 (0.037)	2322 (18)	1.50
**789**	664 (95)	7.810 (0.068)	2328 (10)	0.86
**1000**	994 (94)	7.650 (0.072)	2322 (8)	0.61

Exeriments were conducted at additional target CO_2_ concentrations using certified gas mixtures of 180, 560, 1500 and 1800 ppm for which complete carbonate chemistry is not available.

Following acclimation, individuals were transferred into 0.2 µm-filtered seawater that had been bubbled with the same gas concentration as the acclimation medium. Specimens were contained in glass, gas-tight syringes that served as micro-respirometry chambers. A control syringe with no specimen was incubated simultaneously. All respiration experiments were conducted at −1.8°C. After 12–24 hours, the oxygen concentration was measured in each syringe using a Strathkelvin oxygen electrode in a water-jacketed housing [6]. The oxygen consumption rate was calculated from the difference in oxygen concentration between the animal and control syringes. Following measurement, animals were removed, gently blotted dry and weighed on a Cahn microbalance. The volume of seawater in the chambers was approximately 500x animal mass. Control measurements on seawater that had previously contained an animal revealed no significant microbial respiration.

The starting oxygen concentration in the chambers was 360 ± 5 µM. The concentration at the end of a respiration run was, on average, 262 ± 40.6 µM. Assuming a respiratory quotient (CO_2_ excreted:O_2_ consumed) near 0.7, the respiratory CO_2_ released in the chambers would have gradually reduced the pH over the course of the 12–24 hours experiments by ∼0.2 units. The seawater volumes used in respiration experiments were too small to permit carbonate chemistry measurements in addition to the oxygen and ammonia [6] measurements being made already. However, pH was measured in 2007 following incubations in two experiments at 380 ppm. The pH was reduced following ∼24 hours animal incubation from 8.07 to 7.84 (n  =  2).

While we cannot rule out the possibility that the lower oxygen level or pH experienced toward the end of a respiration run affected our measurements, most marine animals, including those living in relatively high oxygen in the Southern Ocean, are capable of regulating their rate of metabolism to ∼30% saturation [19].

Phytoplankton abundance was estimated from chlorophyll *a* concentrations derived from the Sea-viewing Wide Field-of-view Sensor (SeaWiFS) following previously published methods [6], [7]. Monthly mean chlorophyll images were downloaded from the National Aeronautics and Space Administration (NASA) ocean color website nominally at 4-km resolution for December of each season [22]. The arithmetic mean was calculated for all ice- and cloud-free pixels from 72 to 79°S and 162–170°E, within the vicinity of McMurdo Station where the pteropods were collected (Table 1). Monthly composite images were used in this analysis because pteropods are believed to be long-term integrators of the ecosystem on the scale of weeks to months and because of high levels of cloud cover that obscure the daily imagery.

## Results

**Figure 1:**
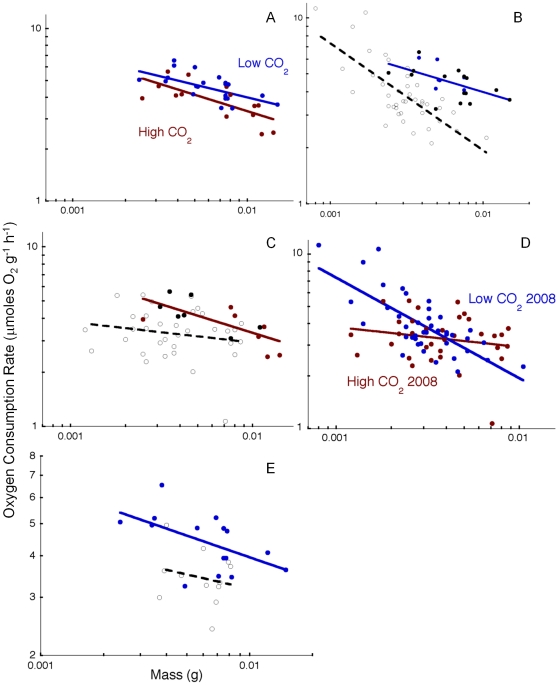
Oxygen consumption rates (MO_2_, µmoles O_2_ g^−^1 h^−1^) of the pteropod, *Limacina helicina forma antarctica* as a function of body mass (M). A) In 2007, MO_2_ was significantly higher at low (380 + 180 µatm, blue; MO_2_  =  1.29 M^−0.25^) than at high (789 + 1000 µatm, red; MO_2_  =  0.78 M^−0.32^) CO_2_ partial pressure (PCO_2_). The individual CO_2_ treatment levels are separated in subsequent panels. MO_2_ was significantly higher in 2007 (closed circles) compared to 2008 at both low (panel B; open circles; MO_2_  =  0.14 M^−0.58^; closed circles180, blue and 380, black, equation above) and high (panel C; open circles, MO_2_  =  1.73 M^−0.12^; closed circles 790, red and 1000, black, equation above) CO_2_ partial pressures. D) In 2008, carbon dioxide (1000 ppm, red, equation above) had no effect on MO_2_ relative to control levels (380 ppm, blue, equation above). E) Food deprivation in the lab (4–6 days, open circles) caused a significant reduction in MO_2_ relative to field-caught specimens in 2007 (380 only, MO_2_  =  1.29 M^−0.25^, closed circles). Significant differences are at p  =  0.05, ANCOVA.

**Figure 2:**
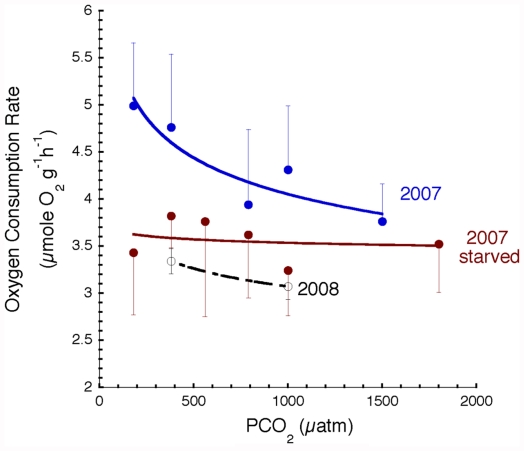
Oxygen consumption rates (MO_2_, µmoles O_2_ g^−1^ h^−1^) of the pteropod, Limacina helicina forma antarctica normalized to a common body mass (5 mg). At low PCO_2_, MO_2_ of fed specimens (blue, fed  =  held less than 2 days prior to acclimation and measurement) are significantly higher than those held in captivity for 4–6 days prior to incubation and measurement (starved, red) in 2007. However, MO_2_ in fed specimens declines strongly with increasing PCO_2_ and MO_2_ is similar between fed and starved specimens at high PCO_2_ (> 1500 µatm). Fed animals from 2008 (open circles, black) have similar rates to specimens starved in 2007 regardless of PCO_2_. Data are means and error bars are standard deviations.

The oxygen consumption rate (MO_2_, µmole O_2_ g^−1^ h^−1^, −2°C) of *Limacina helicina antarctica* was significantly higher at 380 ppm than at 790 ppm (t-test, p  =  0.015). However, this effect is at least partly due to differences in body size range between treatments. The rate of oxygen consumption in animals generally decreases with increasing body mass (M) according to MO_2_  =  b_0_ M*^b^*, where *b* is a scaling coefficient describing the slope of the relationship and b_0_ is the y-intercept for the scaling curves, which varies between species and with treatment effect. However, limited body size range and sample size (Table 1) precluded the analysis of scaling because there was substantial variation in the scaling coefficient measured for each treatment (mean value *b*  =  −0.21±0.05) and the slopes were often not significantly different from zero. Thus, we adopted two approaches to analyze the effects of PCO_2_ on metabolism. First, because there was not a significant difference between either 180 and 380 or between 790 and 1000 ppm treatments in 2007, these were combined as low and high CO_2_ treatments (Figure 1A), respectively, for comparison with measurements at 380 (Figure 1B) and 1000 ppm (Figure 1C, 1D) in 2008. The combined data provided sufficient size range for scaling analysis via ANCOVA. A second approach consisted of normalizing each measurement to a common body mass of 5 mg using an assumed scaling coefficient of −0.25 (Figure 2). This scaling coefficient is considered generally, though not universally, applicable for animals and falls near the mean value for *L. helicina antarctica* and is similar to scaling coefficients reported previously for pteropods [7], [23].

The rates measured at 180–380 µatm (MO_2_  = 1.25 M^−0.25^, p = 0.007) were significantly higher (ANCOVA, p  =  0.004) than those measured at elevated target CO_2_ levels in 2007 (789–1000 µatm,  =  0.78 M^−0.32^, p  =  0.0008; Fig. 1). Furthermore, once normalized to a common body mass (Table 1), MO_2_ declined significantly with increasing PCO_2_ up to 1500 µatm (MO_2_  =  10.02 PCO_2_^−0.13±0.03^, R^2^  =  0.84, p = 0.0017; Fig. 2). However, an effect of CO_2_ on metabolism was observed only in 2007, a year in which we found relatively high concentrations of phytoplankton (3.56 mg Chl m^−3^, Table 1). In 2008, phytoplankton biomass was lower (1.61 mg Chl m^−3^, Table 1) and metabolism was already suppressed (Fig. 2). Previously published (6) rates and Chl *a* levels (from 1999 and 2001, Table 1), as well as laboratory food-deprivation trials described below, support our supposition that food availability is driving the interannual variability in metabolic rate.

A significant reduction in metabolic rate (∼20%) was observed after 4 days in captivity and no further reductions were observed during additional time in captivity. These results are described in detail elsewhere [6]. Positive controls (i.e. animals fed in captivity) could not be conducted because feeding in *L. helicina* involves deployment of a large mucous web that becomes quickly entangled and is abandoned by the animal in captivity. However, studies in gymnosomatous pteropods, which feed in the lab on thecosomatous pteropods, reveal a similar feeding effect on metabolism [6]. In 2007, we compared the effect of PCO_2_ on metabolism in specimens that were measured within 2 days of capture with those that were measured after 4 days of food deprivation in captivity (Figure 1E, 2). MO_2_ at low PCO_2_ in 2007 (b_0_  =  1.25, see full equation above) is higher than at similar CO_2_ levels in 2008 (MO_2_  =  0.14 M^−0.58^; ANCOVA, p  =  0.002; Fig. 2). No effect of CO_2_ was observed in 2008 between control (380 µatm) and treatment (1000 µatm) (Fig. 1D).

The metabolic rates measured under control conditions in seasons with low phytoplankton biomass (2001, 2008) were of similar magnitude to those measured in specimens that were deprived of food in the lab or exposed to high CO_2_ in 2007 (Fig. 2). Thus, the effects of the CO_2_ and regional phytoplankton abundance are not additive. However, food deprivation in the lab caused an additional decrease in metabolism in 2008 [6] suggesting that the low basal rates measured in freshly caught animals were not a simple function of gut emptiness and the absence of specific dynamic action (see below), but rather a plastic response to long-term food supply that influences the response to ocean acidification.

## Discussion

Pteropods have received wide attention as early forecasters of biological impacts of ocean acidification due to their very thin, highly soluble shells [1], [4], [5]. This is especially true in the cold waters of the Southern Ocean where the effects of ocean acidification may first become visible [4], [18] and where pteropods are abundant and trophically important [24], [7]. Early qualitative studies suggested that pteropod shells are susceptible to dissolution under high CO_2_ [4], [5]. However, only a few studies have examined the response of pteropods to high CO_2_ under controlled conditions. Comeau et al. [25] reported that larvae of the thecosomatous pteropod, *Cavolinia inflexa*, show reduced shell growth at a PCO_2_ of 857 ppmv and lack shells completely at much higher CO_2_ levels. In a similar study with the arctic form of *Limacina helicina*, the rate of calcification declined with increasing PCO_2_ in adult individuals [26] but net shell growth was observed even at low aragonite saturation values. The metabolic rate of this species was elevated by high CO_2_, but only at high temperature [26]. This result conflicts with the metabolic suppression reported here (see below). Lischka et al. [27] reported increased shell degradation and reduced shell size and incremental growth in juveniles of Arctic *L. helicina* held without food over 29 days at experimentally elevated PCO_2_ (up to 1150 ppm). The animals from this latter study had begun their over-wintering period and the authors reasoned that feeding was unimportant during this life stage. However our findings suggest that long-term feeding history, not just the gut fullness at the time of the experiment, can influence the response of pteropods to ocean acidification.

Metabolic suppression (∼20%) as a result of low phytoplankton biomass in the Ross Sea, Antarctica, was first recorded for *L. h. antarctica* in 2000–01 [7]. The year following those measurements, *L. h. antarctica* was absent from McMurdo Sound for the first time on record [7]. Food deprivation was hypothesized to have led to poor accumulation of energy reserves that are required for over-winter survival and for reproduction the following spring. The relationship between metabolism and productivity that we’ve shown now over four years (Table 1), suggests that feeding history over long-time scales (i.e., weeks, months and possibly even seasons) plays an important role in pteropod energetics. Little is known about foraging habits of pteropods and the spatial and temporal scales over which they feed in natural conditions. Satellite derived chlorophyll used in this study is only a proxy for food availability and further studies are warranted to elucidate the relationship between foraging scales and local and regional phytoplankton biomass. Gut clearance prior to measurement is an insufficient control on these effects because metabolic rate remains elevated in the absence of food for up to 4 days whereas gut-clearance occurs relatively quickly [6]. Moreover, food deprivation in the lab causes an additional metabolic suppression beyond the low baseline level recorded in years with low phytoplankton concentration, suggesting a plasticity of basal metabolism that responds to feeding history.

Feeding typically elevates metabolism above the basal rate by a factor known as the specific dynamic action (SDA). The extent and duration of the SDA is species-specific and may last from hours to weeks [28]. The metabolic rate of *L. h. antarctica* after 4–13 days without food in the laboratory in 2007 [6] is similar to the suppressed rates reported here for freshly captured specimens under either low phytoplankton concentrations in 2008 or under elevated carbon dioxide levels in 2007. Interestingly, the highest rates measured in *L. h. antarctica* in the present study and previously [7], [23] are lower, by as much as half, than those reported by Comeau et al [25] for the Arctic *L. helicina* population. More importantly, the response to elevated CO_2_ reported by Comeau et al. [25] was in the opposite direction of that observed here. This apparent contrast may result from physiological [29], [30] and genetic [31] differences between the Arctic and Antarctic populations of this supposedly “bipolar” pteropod species. However, it may also be that baseline metabolism is similar between the two populations under similar conditions and that the observed difference in response to CO_2_ results from differences in body size or nutritional and energetic condition. Metabolism in pteropods is very dependent on temperature, lifestyle, body size, and ontogeny [23], [25], [27] as well as seasonal differences in regional productivity and feeding history [6], [7]. Most of these variables were uncontrolled in previous studies, yet all may confound the ability to observe the effects of ocean acidification in pteropods and organisms more generally.

Metabolic suppression, whether induced by food deprivation or high CO_2_, is adaptive in an environment in which phytoplankton biomass is subject to seasonal and natural climate oscillations [32], [33]. However, it is not adaptive under chronic stress such as that expected from ocean acidification or anthropogenic changes to food availability. Anthropogenic warming and ocean acidification are expected to influence both the quantity and quality of phytoplankton available in surface waters via changes in surface irradiance, nutrient availability and sea-ice cover [8], [34]. Along the Antarctic Peninsula, for example, the relative abundance of small phytoplankton has increased in the past decade [35]. Ocean acidification may also alter productivity and phytoplankton species dynamics, favoring large diatoms over *Phaeocystis antarctica*, which is common in the Ross Sea [8]. The type of phytoplankton available, not just total phytoplankton abundance, is known to influence pteropod condition (e.g. lipid composition) with cascading effects on their predators [36]. Global warming and ocean acidification may act directly, or synergistically via changes in food quality and quantity, to alter the energetic status of zooplankton, including pteropods as suggested here.

Our results underscore the inherent difficulties in measuring and, more so, predicting the response of marine organisms to changing environmental conditions. Long-term observations and the inclusion of multiple stressors in analysis of ocean acidification are needed. Conflicting reports on the ecological effects of ocean acidification [37], [38] may reflect the very real complexity of physiological responses to multi-faceted climate change and natural environmental variability. However, given the potential importance of the CO_2_-response of key species such as *L. helicina antarctica*, it is imperative that we understand the environmental variables that moderate the response to ocean acidification as well as the energetic consequences. A mechanistic understanding of species- and environment-specific responses is a daunting, but necessary, goal if we hope to understand the consequences of ocean acidification at the ecosystem level. *Limacina helicina* is a key grazer in polar waters, an important food source at several trophic levels, and plays a role in the biogeochemical cycles of the Southern Ocean [24]. We’ve shown here that ocean acidification and associated environmental changes can induce a sustained metabolic suppression that, in the absence of acclimation or adaptation, will have consequences for the fitness of this species.
